# Risk of placental abruption in relation to migraines and headaches

**DOI:** 10.1186/1472-6874-10-30

**Published:** 2010-10-26

**Authors:** Sixto E Sanchez, Michelle A Williams, Percy N Pacora, Cande V Ananth, Chungfang Qiu, Sheena K Aurora, Tanya K Sorensen

**Affiliations:** 1 Department of Obstetrics and Gynecology, Hospital Nacional dos de Mayo, & Universidad San Martin de Porres, Lima, Peru; 2 Department of Epidemiology, University of Washington School of Public Health, Seattle, WA, USA; 3 Center for Perinatal Studies, Swedish Medical Center, Seattle, WA, USA; 4 Department of Obstetrics and Gynecology, Hospital Nacional Docente Madre Niño San Bartolomè; & Universidad Nacional Mayor de San Marcos, Lima, Peru; 5 Division of Epidemiology and Biostatistics; Department of Obstetrics, Gynecology, and Reproductive Sciences UMDNJ-Robert Wood Johnson Medical School, New Brunswick, NJ, USA; 6 Swedish Headache Center, Seattle WA, USA

## Abstract

**Background:**

Migraine, a common chronic-intermittent disorder of idiopathic origin characterized by severe debilitating headaches and autonomic nervous system dysfunction, and placental abruption, the premature separation of the placenta, share many common pathophysiological characteristics. Moreover, endothelial dysfunction, platelet activation, hypercoagulation, and inflammation are common to both disorders. We assessed risk of placental abruption in relation to maternal history of migraine before and during pregnancy in Peruvian women.

**Methods:**

Cases were 375 women with pregnancies complicated by placental abruption, and controls were 368 women without an abruption. During in-person interviews conducted following delivery, women were asked if they had physician-diagnosed migraine, and they were asked questions that allowed headaches and migraine to be classified according to criteria established by the International Headache Society. Logistic regression procedures were used to calculate odds ratios (aOR) and 95% confidence intervals (CI) adjusted for confounders.

**Results:**

Overall, a lifetime history of any headaches or migraine was associated with an increased odds of placental abruption (aOR = 1.60; 95% CI 1.16-2.20). A lifetime history of migraine was associated with a 2.14-fold increased odds of placental abruption (aOR = 2.14; 95% CI 1.22-3.75). The odds of placental abruption was 2.11 (95% CI 1.00-4.45) for migraineurs without aura; and 1.59 (95% 0.70-3.62) for migraineurs with aura. A lifetime history of tension-type headache was also increased with placental abruption (aOR = 1.61; 95% CI 1.01-2.57).

**Conclusions:**

This study adds placental abruption to a growing list of pregnancy complications associated with maternal headache/migraine disorders. Nevertheless, prospective cohort studies are needed to more rigorously evaluate the extent to which migraines and/or its treatments are associated with the occurrence of placental abruption.

## Background

Placental abruption, the premature separation of the placenta before delivery of the fetus, complicates approximately 1% of all pregnancies [1-3]. Placental abruption is a significant cause of maternal and neonatal morbidity and infant mortality. Maternal complications include hemorrhagic shock, coagulopathy, disseminated intravascular coagulation, and renal failure. The condition is also associated with increased risks of preterm delivery and intrauterine growth restriction. Neonatal death and long term complications are also adverse outcomes following placental abruption [4,5]. Although placental abruption accounts for a significant proportion of maternal and fetal morbidity and mortality, the etiology of this important obstetrical complication remains largely speculative. Evidence from studies conducted during the last three decades, however, suggest that hypertensive disorders, increased maternal age, grand-multiparity, thrombophilia, cigarette smoking, illicit drug use, particularly cocaine, and external trauma to the abdomen are associated with an increased risk of placental abruption [2,5-7]. Other putative risk factors include maternal iron deficiency anemia [8], maternal psychiatric disorders including depression [9], hyperhomocystinemia [10], as well as maternal infection and/or inflammation [11]. Results from molecular epidemiology studies suggest that variants in genes in selected pathways (e.g., coagulation, fibrinolysis, platelet function, infection/inflammation, angiogenesis, and the renin-angiotensin systems) may also be important genomic risk factors of placental abruption [12-14]. On balance, available epidemiological and clinical data suggest that vascular dysfunction manifesting as impaired uteroplacental perfusion may be of etiologic importance in placental abruption.

Migraine, a common chronic-intermittent neurovascular headache disorder, is ranked among the world's twenty most disabling medical conditions by the World Health Organization [15]. Migraine is characterized by episodic severe headache accompanied by autonomic nervous system dysfunction. Some patients with migraine have headaches that are accompanied by transient neurological symptoms and are thus classified as having migraine with aura [16]. Women are more commonly affected than men, with reported lifetime prevalence estimates of 16-32% for women and 6-9% for men [17]. Migraine risk varies considerably across the life course and is most prevalent among women during their childbearing years [18,19]. Associations between migraine and vascular disease have long been hypothesized and considered over the last century, but results reported in the epidemiologic literature are contradictory. However, evidence from more recent rigorously designed, conducted and analyzed studies now suggest consistent relationships between migraine and vasospastic disorders such as variant angina and Raynaud's phenomenon, as well as ischemic stroke in young women [20,21]. A recent population-based cross-sectional study of Dutch adults found that female migraineurs were at increased risk of subclinical brain infarcts and white matter lesions (OR = 2.1; 95% CI, 1.0-4.7), which may increase the risk of future stroke and dementia [22].

After decades of believing that migraine had no adverse effects on pregnancy and parturition [23,24], a conclusion based on relatively few studies with conflicting results, more recent studies have documented elevated risks of preterm delivery [25], low birth weight [25] and pregnancy-induced hypertension and preeclampsia among women with migraine [19,25-28]. To date, however, no investigators have evaluated the association between migraine and placental abruption risk. Because emerging evidence suggest that placental abruption is an ischemic placental disease with epidemiological and pathophysiological characteristics similar to preterm delivery, intrauterine growth restriction and preeclampsia [2,29], we hypothesized that pregnant migraineurs, compared with those without the condition would have an elevated risk of placental abruption. To test this hypothesis, we analyzed data from a large case-control study of placental abruption conducted in Lima, Peru.

## Methods

### Study population

This case-control study was conducted at the Hospital Nacional dos de Mayo, Instituto Especializado Materno Perinatal, Hospital Edgardo Rebagliati Martins, Hospital Nacional Hipolito Unanue, and Hospital Nacional Docente Madre Niño San Bartolomè in Lima, Peru, from September 2006 through September 2008. This study was approved by the institutional review board of each participating institution. All participants provided written informed consent.

Placental abruption cases were identified by daily monitoring of all new admissions to antepartum, emergency room, and labor and delivery wards of participating hospitals. Study subjects were recruited during their hospital stay. Hospital medical records were reviewed so that clinical diagnostic signs, symptoms and physical characteristics of placental abruption could be objectively confirmed; and so that other clinical diagnoses associated with late pregnancy vaginal bleeding could be excluded. During the study period, there were an estimated total of 55,802 deliveries of which 452 were complicated by placental abruption. Twenty-eight cases were missed because of inadequate staffing. Of the remaining 424 cases approached, 382 (90%) elected to participate in the study. The diagnosis of placental abruption was based on routine clinical examination performed by the attending physician. For the diagnosis of placental abruption, we required evidence of blood clot (retroplacental clots) or bleeding behind the placenta accompanied by at least 2 of the following signs and symptoms: 1) vaginal bleeding in late pregnancy that was not associated with placenta previa or cervical lesions; 2) uterine tenderness and/or abdominal pain; and 3) fetal distress or death. Controls were selected from eligible women who delivered at the participating hospitals during the study period. Following the recruitment of a placental abruption case, a control patient was sought for recruitment from the same hospital. Potential controls were identified by reviewing daily delivery logs in participating hospitals. Eligible controls were women who did not have a diagnosis of placental abruption and whose medical record review later confirmed this fact. Of the 429 controls approached, 86% (n = 369) agreed to participate in the study.

### Data collection and variable specification

We used a standardized, structured Spanish-language questionnaire to collect information regarding maternal sociodemographic, medical, reproductive, and lifestyle characteristics during in-person interviews. All interviews were conducted in the hospital by trained research interviewers. All interviews were conducted in Spanish. Information collected during the interviews included maternal age, marital status, employment status during pregnancy, medical history, and smoking and alcohol consumption during pregnancy.

Information about headache and migraine symptoms were collected using the decode Genetics migraine questionnaire [30] which was translated to Spanish for use in Lima, Peru. Women were asked whether they had ever experienced headache attacks. For those answering yes, separate questions were asked about (*i*) age of onset of first attack, (*ii*) timing of last attack; (*iii*) typical duration of attacks; *iv*) whether they had ever been diagnosed as having migraine by a doctor; and (*v*) age at diagnosis. Women were also asked to indicate characteristics symptoms associated with their headaches. These included location of pain, visual disturbances including flares or zigzag patterns, sensory disturbances including progressive numbness over the face and body, difficulty with speech, inhibition of daily activity, and triggers of headaches. Collected information allowed us to classify women for several headache and migraine variables. Women were classified as ever having headache before (yes, no) or during pregnancy (yes, no). Those women with headaches were classified according to the timing of onset (never, final month of pregnancy, first 8 months of pregnancy and prior to pregnancy) relative to the index pregnancy. We classified women according to whether they had been diagnosed, by a physician, as having migraine (yes, no). We also were able to classify women based on the modified International Classification of Headache Disorders-II (ICHD-II) criteria for migraine [31]. "Strict Migraine" (ICHD-II category 1.1 or 1.2) was defined by at least 5 lifetime headache attacks lasting 4-72 hours, with at least 2 of the qualifying pain characteristics (unilateral location, pulsating quality, moderate or severe pain intensity, aggravation by routine physical exertion), at least one of the associated symptoms (nausea and/or vomiting, photo/phonophobia), and not readily attributable to another central nervous system disorder or head trauma (according to subject self-report). "Probable Migraine" (ICHD-II category 1.6) was designated if all but one of the strict migraine criteria were fulfilled, excluding headaches attributable to another disorder. Subjects classified as strict or probable migraine were combined for further analysis. We included both "Strict Migraine" and "Probable Migraine" as migraineurs for these analyses. Migraineurs were further classified as "migraine with aura" and "migraine without aura" according to ICHD-II diagnostic criteria [31]. Women with non-migrainous headaches were classified as having "Tension-type Headaches" or "Other Headaches" according to ICHD diagnostic criteria.

We used the Patient Health Questionaire-9 (PHQ-9) to assess participants' experience of depression or depressive symptoms during pregnancy. The instrument has been demonstrated to be a reliable tool for assessing recent psychosocial stressors among obstetrics-gynecology patients [32] and in Spanish-speaking women [33]. In a recent validation study of the PHQ-9 questionnaire, the authors concluded that the instrument is a reliable and valid measure of depression severity and a useful clinical and research tool [34]. The PHQ-9 scale includes nine items, and choices for responses were a) never; b) several weeks over the pregnancy; c) more than half the pregnancy; or d) nearly the whole pregnancy. The PHQ-9 total score is the sum of scores for the nine items for each woman, and ranged from 0-27. We categorized participants as exhibiting symptoms consistent with a diagnosis of moderate or severe depression (PHQ-9 score ≥ 10) [34].

Maternal and infant records were reviewed to collect detailed information concerning antepartum, labor, and delivery characteristics, as well as conditions of the newborn. Maternal anthropometric measures (height, weight, and mid-arm circumference) were taken during participants' hospital stays. Gestational age was based on the date of the last menstrual period and was confirmed by an ultrasound examination before 20 weeks. Pre-pregnancy body mass index (BMI), a measure of overall maternal adiposity, was calculated as (self-reported) weight in kilograms divided by height in meters squared. Women were classified as lean (BMI < 19.8 kg/m^2^), normal (BMI = 19.8-26.0 kg/m^2^), overweight (BMI = 26.1-29.0 kg/m^2^) or obese (BMI > 29.0 kg/m^2^).

### Analytical population

The analytical population for the study is derived from the 382 placental abruption cases and 369 controls enrolled in the study. For the purposes of the present study, 8 women with twin or higher-order pregnancies (7 cases and 1 control) were excluded, leaving 375 placental abruption cases and 368 controls for analysis.

### Statistical analysis

We examined the frequency distribution of maternal socio-demographic characteristics and reproductive histories according to case and control status. Initial bivariate analyses were carried out to determine unadjusted odds ratio (OR) and 95% confidence interval (CI). Effect modification was evaluated by stratified analyses and by including appropriate interaction terms in logistic regression models [35]. On the basis of a prior literature [6,7,9], we conducted exploratory analyses to evaluate the extent to which observed associations between placental abruption and migraine status were modified by maternal age, pre-pregnancy overweight status, depression, and hypertensive status. If there appeared to be no effect modification (p-value for cross-product term was > 0.05), logistic regression procedures were used to simultaneously control for confounding variables while estimating ORs and 95% CIs.

We selected potential confounders from a list of variables for which there is evidence of possible associations with migraine and placental abruption (from prior published studies) and that met criteria for confounding based on a review of the literature and assessment of potential causal relationships based on prior knowledge. We then controlled for potential confounders that changed multivariable ORs by ≥ 10% relative to the unadjusted OR (35). Final logistic regression models included confounders, as well as those covariates of *a priori *interest (i.e., maternal age, maternal educational attainment, maternal smoking status and alcohol consumption). Maternal employment status, parity, pre-pregnancy body mass index, and prenatal vitamins use were not found to be confounders and thus were not included in final models. All continuous variables are presented as mean ± standard deviation (SD). All reported p-values are two-tailed.

Prior to initiating the study, we estimated that a study size of 300 cases and an equal number of controls would be sufficient (> 80% power) for estimating odds ratios of ≥ 2.0 if exposure frequencies were ≥ 10%, and if significance was set at 0.05. All analyses were performed using STATA 9.0 statistical software (Stata, College Station, Texas, USA).

## Results

Forty-nine participants (37 cases, 12 controls) were classified as having "Strict Migraine"; and 18 participants (7 cases, 11 controls) were classified as having "Probable Migraine". Migraineurs were further classified as "migraine with aura" (n = 32) and "migraine without aura" (n = 35) according to ICHDII diagnostic criteria (31). Socio-demographic and reproductive characteristics of placental abruption cases and controls are presented in Table 1. Cases and controls were similar with regards to maternal age, parity, marital status and educational attainment. Compared with controls, cases were less likely to have received prenatal care. As expected, placental abruption cases were more likely to deliver preterm and low birth weight infants and to have a history of hypertensive disorders. Approximately 15.7% of placental abruption cases delivered stillborn infants; there were no stillbirth among controls.

**Table 1:** Socio-Demographic and Reproductive Characteristics and Infant Outcomes in the Study Population, Lima, Peru, 2006-2008

Characteristics	Placental Abruption				p-value
		
	Cases (N = 375)		Controls (N = 368)		
	n	%	n	%	
Maternal Age at Delivery (years)	28.4 ± 6.7^1^		28.0 ± 6.3		0.38
Maternal Age at Delivery (years)					
< 20	39	10.4	30	8.2	0.30
20-29	174	46.4	191	51.9	
30-34	86	22.9	78	21.2	
≥ 35	75	20.0	67	18.2	
Missing	1	0.3	2	0.5	
Parity	1.32 ± 1.50^1^		1.46 ± 1.51		0.19
0	161	42.9	146	39.7	0.63
1-2	143	38.1	145	39.4	
≥ 3	71	18.9	77	20.9	
≤ High School Education	241	64.3	216	58.7	0.29
Employed during Pregnancy	186	49.6	176	47.8	0.66
Planned Pregnancy	133	35.5	115	31.3	0.47
No Prenatal Care	41	10.9	22	6.0	0.02
No Prenatal Vitamin	57	15.2	45	12.2	0.38
Smoking during Pregnancy	8	2.1	6	1.6	0.48
Alcohol use during Pregnancy	35	9.3	30	8.2	0.67
Illicit Drug use during Pregnancy	2	0.5	1	0.3	- - -
Depressive Symptoms^3^	33	8.8	10	2.7	< 0.001
Pre-pregnancy Body Mass Index (kg/m^2^)	23.7 ± 3.7^1^		23.4 ± 3.5		0.31
Pre-pregnancy Body Mass Index (kg/m^2^)					
< 19.8	39	10.4	38	10.3	0.38
19.8-26.0	227	60.5	245	66.6	
26.1-29.0	47	12.5	36	9.8	
≥ 29.0	27	7.2	25	6.8	
Missing	35	9.3	24	6.5	
Chronic Hypertension	20	5.3	10	2.7	0.07
Preeclampsia or Eclampsia	98	26.1	11	3.0	< 0.001
Previous History of Placental Abruption	2	0.9	1	0.5	0.62
Infant Birth Weight (grams)^2^	2438 ± 860^1^		3306 ± 502		< 0.001
Low Birth Weight Infant (< 2500 grams)^2^	196	52.3	17	4.6	< 0.001
Gestational Age at Delivery (weeks)^2^	35.3 ± 3.8^1^		38.7 ± 1.8		< 0.001
Preterm Delivery Infant (< 37 weeks)^2^	204	54.4	20	5.4	< 0.001
C-Section Delivery	357	95.3	144	39.1	< 0.001
Stillbirth Delivery	59	15.7	0	0.0	- - -

^1^Mean ± SD (SD: standard deviation)

^2^Restricted to live birth only (314 cases and 368 controls)

^3^PHQ-9 depression symptom score ≥ 10, consistent with moderate or severe depression

The lifetime prevalence of headaches or migraine was 40.0% for placental abruption cases and 29.6% for controls (Table 2). After adjusting for maternal age, prenatal care enrollment and maternal depression status, maternal lifetime history of headaches or migraine was associated with an increased odds of placental abruption (aOR = 1.60; 95% CI 1.16-2.20). A lifetime history of migraine was associated with a 2.14-fold increased odds of placental abruption (aOR = 2.14; 95% CI 1.22-3.75). The odds of placental abruption was 2.11 (95% CI 1.00-4.45) for migraineurs without aura; and 1.59 (95% 0.70-3.62) for migraineurs with aura. A lifetime history of tension-type headache was also increased with placental abruption (aOR = 1.61; 95% CI 1.01-2.57). Other non-migrainous headaches were only marginally and statistically nonsignificantly related with the odds of placental abruption (aOR = 1.35; 95% CI 0.87-2.09). When reports of migraine were grouped according to when they started, the association with placental abruption was evident for migraine with onset during pregnancy (aOR = 2.63; 95% CI 0.48-14.44) and migraine with onset prior to pregnancy (aOR = 1.79; 95% CI 0.98-3.27) although these associations did not reach statistical significance.

**Table 2:** Unadjusted and Adjusted Odds Ratio (OR) and 95% Confidence Interval (CI) for Placental Abruption in Relation to Maternal History of Headaches and Migraine, Lima, Peru, 2006-2008

	Placental Abruption					
			
	Cases (N = 375)		Controls (N = 368)			
Exposure Status	n	%	n	%	Unadjusted OR (95% CI)	^1^Adjusted OR (95% CI)
**Lifetime History of Headaches and Migraine (ICHD Criteria)**						
No	225	60.0	259	70.4		
Yes	150	40.0	109	29.6	1.58 (1.17-2.15)	1.60 (1.16-2.20)
*Migraine headache*	*44*	*11.7*	*23*	*6.3*	*2.20 (1.29*-*3.76)*	*2.14 (1.22*-*3.75)*
*Tension-type headaches*	*51*	*13.6*	*38*	*10.3*	*1.54 (0.98*-*2.44)*	*1.61 (1.01*-*2.57)*
*Other headaches*	*55*	*14.7*	*48*	*13.0*	*1.32 (0.86*-*2.02)*	*1.35 (0.87*-*2.09)*
**Lifetime History of Migraine with aura(ICHD criteria)^2^**						
No Headaches or Migraine	225	60.0	259	70.4	1.00 (referent)	1.00 (referent)
*Migraine without aura*	*23*	*6.1*	*12*	*3.3*	*2.21 (1.07*-*4.53)*	*2.11 (1.00*-*4.45)*
*Migraine with aura*	*21*	*5.6*	*11*	*3.0*	*2.20 (1.04*-*4.66)*	*1.59 (0.70*-*3.62)*
**Onset of Migraine (ICHD criteria)^2^**						
No Headaches or Migraine	225	60.0	259	70.4	1.00 (referent)	1.00 (referent)
*Yes, onset during pregnancy*	*5*	*1.3*	*2*	*0.5*	*2.88 (0.55-14.98)*	*2.63 (0.48-14.44)*
*Yes, onset prior to pregnancy*	*39*	*10.4*	*21*	*5.7*	*2.14 (1.22-3.74)*	*1.79 (0.98-3.27)*
**Headache or Migraine Onset**						
No headache	225	60.0	259	70.4	1.00 (referent)	1.00 (referent)
Final month of pregnancy	5	1.3	3	0.8	1.92 (0.45-8.12)	1.67 (0.39-7.24)
First 8 months of pregnancy	34	9.1	24	6.5	1.63 (0.94-2.83)	1.62 (0.92-2.85)
Prior to pregnancy	111	29.6	82	22.3	1.56 (1.11-2.18)	1.66 (1.16-2.37)
**History of Physician Diagnosed Migraine**						
No	350	93.3	348	94.6	1.00 (referent)	1.00 (referent)
Yes	25	6.7	20	5.4	1.24 (0.68-2.28)	1.13 (0.60-2.12)

^1^Adjusted by maternal age, education, smoking status, alcohol consumption, prenatal care utilization and maternal depression status

^2^Women with non-migrainous headache disorders were deleted from this analysis; column percentages will not sum to 100%

The prevalence of physician-diagnosed migraine was low in this study population (6.7% of placental abruption cases and 5.4% of controls). History of physician-diagnosed migraine was associated with a modest but non-significant increased odds of placental abruption (OR = 1.24; 95% CI 0.68-2.28). The association was further attenuated after adjusting for confounding by maternal age, use of prenatal care, and depression status (aOR = 1.13; 95% CI 0.60-2.12).

Inferences from this analysis are limited by the small numbers of women with physician-diagnosed migraine.

We found no evidence of effect modification by maternal pre-pregnancy overweight status or advanced maternal age. We did however, find some evidence suggestive of effect modification by maternal depressive symptoms (Table 3) and by maternal hypertensive status (Table 4). It appeared that women with migraine or headache disorders and depression had a greatly increased odds of placental abruption (aOR = 14.98; 95% CI 1.91-117.39) (Table 3). Similarly, women with migraine and hypertensive disorders had a substantially increased odds of placental abruption (aOR = 13.83; 95% CI 3.13-61.11) (Table 4). Inferences from these exploratory analyses, however, were limited by the small numbers of participants with co-morbid migraine and depression and co-morbid migraine/headaches and hypertensive disorders.

**Table 3:** Unadjusted and Adjusted Odds Ratio (OR) and 95% Confidence Interval (CI) for Placental Abruption in Relation to Maternal History of Headaches/Migraine and Depression Status, Lima, Peru, 2006-2008

Headaches/Migraine & Depression Status	Placental Abruption		^1^Adjusted OR (95% CI)
		
	Cases (N = 375) n	Controls (N = 368) n	
No Headaches/Migraine & No Depression	213	257	1.00 (referent)
Yes Headaches/Migraine & No Depression	32	22	1.86 (1.03-3.34)
No Headaches/Migraine & Yes Depression	12	2	7.31 (1.01-33.31)
Yes Headaches/Migraine & Yes Depression	12	1	14.98(1.91-117.39)
*P-value for the multiplicative interaction term*			*0.31*

Two cases excluded because of missing depression information

^1^Adjusted by maternal age, education, smoking status, alcohol consumption and prenatal care utilization

**Table 4:** Unadjusted and Adjusted Odds Ratio (OR) and 95% Confidence Interval (CI) for Placental Abruption in Relation to Maternal History of Headaches/Migraine and Hypertensive Status, Lima, Peru, 2006-2008

Headaches/Migraine & Depression Status	Placental Abruption		^1^Adjusted OR (95% CI)
		
	Cases (N = 375) n	Controls (N = 368) n	
No Hypertension & No Depression	168	248	1.00 (referent)
Yes Hypertension/Migraine & No Depression	26	21	1.91 (1.03-3.56)
No Hypertension/Migraine & Yes Depression	57	11	7.46 (3.77-14.75)
Yes Hypertension/Migraine & Yes Depression	18	2	13.83 (3.13-61.11)
*P-value for the multiplicative interaction term*			*0.92*

^1^Adjusted by maternal age, education, smoking status, alcohol consumption and prenatal care utilization

## Discussion

To the best of our knowledge, this is perhaps the first study to report the associations of headaches and migraine and placental abruption risk. We found that women with migraine had an increased odds of placental abruption. Because this study may be one of the first to investigate whether maternal migraine status is associated with placental abruption, our findings can only tentatively be compared with studies that have investigated other perinatal outcomes.

Migraine has been associated with a number of adverse reproductive outcomes [16,25-27]. Our findings are generally consistent with other studies reporting associations of migraine with adverse pregnancy outcomes including preterm birth [25], low birthweight [26] and preeclampsia [25-28]. In their population-based study of Taiwanese women, Chen et al. [25] reported increased risks of preterm birth (aOR = 1.24; 95% CI 1.13-1.39) and low birthweight (aOR = 1.16; 95% CI 1.03-1.31) deliveries among mothers with migraine as compared with non-migraineurs. Stronger associations between maternal migraine status and risk of low birthweight deliveries (aOR = 1.84; 95% CI 0.90-3.77) were reported from a study of Italian women [26]. Facchinetti et al [26], recently reported from their prospective cohort study of 702 normotensive pregnant women that those with migraine had a 2.85-fold increased risk of developing hypertensive disorders of pregnancy (aOR = 2.85; 95% CI 1.40-5.81), as compared with non-migraineurs. These findings corroborate results from earlier case control studies conducted in Canada, the US, and Peru [27,28]. Collectively, these studies suggest that risks for adverse perinatal outcomes are increased with maternal prior diagnosis of migraine. Notably, the association between placental abruption and migraine are of similar magnitudes to associations reported previously for placental abruption, such as diabetes mellitus, advanced maternal age, and cigarette smoking during pregnancy [12].

This study has several strengths, including the relatively large sample of placental abruption cases and controls, the fact that a well structured standardized questionnaire was used to collect information suitable for classifying migraine according to established ICHD-II diagnostic criteria [31], and we were able to estimate placental abruption risk according to the timing of migraine onset (i.e., prior to or during pregnancy). The high participation rates for cases and controls (90% and 86%) also served to attenuate concerns about selection bias. Several limitations, however, should be considered when interpreting study findings. First, the case-control study design and our reliance on self-reported signs and symptoms of migraine and headaches (using a questionnaire that was not validated in the specific study population) raises concerns about recall bias. To help mitigate the likelihood of systematic reporting errors, well-trained interviewers used a standard questionnaire to collect information from study participants. Moreover, neither the interviewers nor the participants were aware of any of the specific study hypotheses. Random misclassification (i.e., unrelated to placental abruption case-control status) of the onset, timing and intensity of symptoms, however, may have occurred due to recall error. Such errors in recall may have led to an underestimation of the odds ratios. Nevertheless, prospective cohort studies that allow for clinical confirmation of participants' migraine status are needed to confirm or refute our study findings. Second, we had no information about maternal use of medications to treat migraine. Future studies will have to include this information so that independent and joint effects of maternal migraine and medication use on placental abruption can be evaluated. Additionally, despite controlling for potential confounders, residual confounding by factors not measured in our study (e.g., thrombophilia and maternal use of triptan and ergotamine medications) may have influenced reported risk estimates. Prospective studies are needed to enhance causal inferences concerning associations between placental abruption and migraine.

The possible association between maternal migraine and increased placental abruption risk may be explained by the vascular disorders common to these two pathological conditions. Though not considered a vascular disorder, *per se*, migraine is associated with ischemic stroke [36] and a cardiovascular risk profile [37-39] including chronic systemic inflammation [38,40], abnormal vascular reactivity [41], increased platelet aggregation [42], alterations in magnesium and calcium metabolism and signaling [43], and an imbalance in the synthesis and release of thromboxane and prostacyclin that favors vasoconstriction [44]. Furthermore, identification of a migraine-specific gene at chromosome 19p13, known to be associated with missense mutations in the brain-specific P/Q-type Ca2+ channel alpha1-subunit gene, CACNA1A [45], raises the possibility that genetic regulators of calcium homeostasis may contribute to determining migraine susceptibility. Of note, many of the pathophysiological features of migraine overlap with those commonly noted in pregnancies complicated by placental abruption and other related perinatal disorders including preeclampsia and preterm delivery. Finally, investigators have speculated that migraine that persists during the second and third trimesters in pregnancy may be in part attributable to impaired placental function [26] and synthesis of hormones/opioids that generally account for the attenuation of migraine symptoms normally observed during pregnancy [46]. Evidence from placental ultrasound studies [47], and from those that document alterations in the synthesis and release of placental hormones [48] and other biomarkers [49] in placental abruption cases versus control pregnancies supports this hypothesis.

## Conclusions

Our results suggest that the risk of placental abruption is increased in women having migraine prior to or during pregnancy. Prospective cohort studies, however, are needed to more rigorously evaluate the extent to which migraine and/or its treatments are associated with the occurrence of placental abruption. Results from studies that allow for characterizing migraine history according to age of onset, frequency, and triggers of migraine episodes will likely yield new information that can be used to develop strategies for the prevention and control of migraine and placental abruption in reproductive-aged women. Our results add to the evolving literature that suggests that pregnant migraineurs should be considered at high risk for developing pregnancy complications [16,25,26]. Preconception counseling, important for women with any medical conditions including migraine, may provide opportunities for optimizing control of migraine symptoms with the lowest effective doses of the lowest number of medications; or if appropriate, with non-pharmacological treatment including biofeedback-assisted relaxation, hydration, improved sleep hygiene and reductions in occupational and home activities.

## Competing interests

The authors declare that they have no competing interests.

## Authors' contributions

SES and MAW had full access to all the data in the study and take responsibility for the integrity of the data, the accuracy of the data analysis, and the decision to submit for publication. MAW conceived, designed and obtained funding for the study. MAW, SES and CQ, analyzed the data. SES, MAW and CVA, drafted the manuscript. All authors (i.e., SES, MAW, PNP, CVA, CQ, SKA, TKS) interpreted the data, critically revised the draft for important intellectual content, and gave final approval of the manuscript to be published. All of the authors meet the criteria for authorship.

## Pre-publication history

The pre-publication history for this paper can be accessed here:

http://www.biomedcentral.com/1472-6874/10/30/prepub
